# Incidence and outcomes of hospitalized acute ischemic stroke patients with subsequent ST-segment-elevation myocardial infarction

**DOI:** 10.3389/fcvm.2025.1630805

**Published:** 2025-10-30

**Authors:** Arhum Mahmood, Song Peng Ang, Yusuf Kamran Qadeer, Hafeez Ul Hassan Virk, Jonathan Alan Tangsrivimol, Iqra Riaz, Zhen Wang, Mahboob Alam, Markus Strauss, Chayakrit Krittanawong

**Affiliations:** ^1^ Department of Medicine, Henry Ford Hospital, Detroit, MI, United States; ^2^ Division of Internal Medicine, Rutgers Health Community Medical Center, New Brunswick, NJ, United States; ^3^ Division of Cardiology, Department of Medicine, Henry Ford Hospital, Detroit, MI, United States; ^4^ Harrington Heart & Vascular Institute, Case Western Reserve University, University Hospitals Cleveland Medical Center, Cleveland, OH, United States; ^5^ Department of Neurosurgery, The Loyal and Edith Davis Neurosurgical Research Laboratory, Barrow Neurological Institute, St. Joseph's Hospital and Medical Center, Phoenix, AZ, United States; ^6^ Division of Neurosurgery, Department of Surgery, Chulabhorn Hospital, Chulabhorn Royal Academy, Bangkok, Thailand; ^7^ Department of Medicine, Adena Regional Medical Center, Chillicothe, OH, United States; ^8^ Robert D. and Patricia E. Kern Center for the Science of Health Care Delivery, Mayo Clinic, Rochester, MN, United States; ^9^ Division of Health Care Policy and Research, Department of Health Sciences Research, Mayo Clinic, Rochester, MN, United States; ^10^ The Texas Heart Institute, Baylor College of Medicine, Houston, TX, United States; ^11^ Department of Cardiology I- Coronary and Peripheral Vascular Disease, Heart Failure Medicine, University Hospital Muenster, Muenster, Germany; ^12^ Department of Cardiology, Faculty of Health, School of Medicine, University Witten/Herdecke, Witten, Germany; ^13^ HumanX, Delaware City, DE, United States

**Keywords:** stroke, acute ischeamic stroke, myocardial infarction, ST-segment-elevation myocardial infarction, stroke patient care

## Abstract

**Background:**

Patients admitted with acute ischemic stroke (AIS) may experience accompanying acute ST-Segment myocardial infarction after AIS. The cardiovascular risks, incidence, complications, and outcomes of acute STEMI in patients hospitalized with AIS remains underexplored.

**Methods:**

We evaluated 2,804,819 patients that presented with AIS who were listed in the National Inpatient Sample from 2016 to 2021. AIS and STEMI were defined according to the ICD-10 Diagnostic Codes. Patients with Non-STEMI were excluded. The risk of specific complications and outcomes were expressed as percentages. Multivariable logistic regression analysis was used to examine the association of STEMI with a primary outcome of mortality and secondary outcomes. The temporal trend of both the incidence of STEMI after AIS as well as the mortality rate between 2016 and 2021 were expressed as percentages over time.

**Results:**

Of the total (*n* = 2,804,819) patients with AIS, 6,550 also had STEMI diagnosed during the hospitalization. Of these, 1,635 (24.96%) died in the STEMI group and 86,810 (3.10%) died in the group without STEMI. All of the secondary outcome measures were significantly associated with a diagnosis of STEMI. STEMI was associated with mortality [OR 7.43 (95% CI, 6.44–8.57); *P* < 0.001], cardiogenic shock [OR 29.64, (95% CI, 22.64–38.81); *P* < 0.001], cardiac arrest [OR, 7.76 (95% CI, 6.01–10.03); *P* < 0.001], and AKI [OR 1.96 (95% CI, 1.72–2.23); *P* < 0.001] among other complications. When assessed yearly, the temporal trend of STEMI among AIS patients showed a decrease in frequency from about 0.3% in 2016 to about 0.2% in 2021. Furthermore, comparing the mortality between AIS patients with and without STEMI showed a significant difference with a higher mortality in the AIS with STEMI population.

**Conclusions:**

Patients admitted with acute ischemic stroke who had STEMI have a significant mortality increase compared to those who did not have STEMI. They also had a significant increase in secondary complications including cardiac arrest, cardiogenic shock, AKI, and need for further medical interventions. Temporally, we have seen a decrease in STEMI after AIS over the interval.

## Introduction

Stroke and ST-segment elevation myocardial infarction (STEMI) are two leading causes of death and disability worldwide. Epidemiologically, both conditions are driven by atherosclerosis and share several risk factors, including hypertension, diabetes, smoking, and hyperlipidemia. The global burden of stroke remains high, with an estimated 12.2 million new strokes each year, leading to 6.5 million deaths annually. Similarly, STEMI contributes significantly to the overall burden of ischemic heart disease, with thousands of new cases reported each year. In the United States alone, there are approximately 800,000 strokes and 900,000 STEMI events annually.

Despite sharing common pathophysiological mechanisms, including atherosclerosis, thrombosis, and inflammation, the interplay between these two conditions when they occur concurrently remains poorly understood because although, both stroke and myocardial infarction have been studied in isolation, the intersection of these two critical events presents unique challenges that are not adequately addressed by current clinical practices. Moreover, the absence of robust contemporary data impedes efforts to fully understand the changing epidemiology of this overlap, the associated risk factors, and the optimal therapeutic strategies.

Given the paucity of contemporary data, our objective was to investigate the trends, clinical characteristics, and outcomes of patients hospitalized with AIS, stratified by the presence of STEMI.

## Methods

This retrospective cohort study used the National Inpatient Sample (NIS), the largest publicly available all-payer inpatient database in the United States, which is part of the Healthcare Cost and Utilization Project (HCUP). The NIS provides a 20% stratified sample of U.S. hospital discharges and allows for the generation of nationally representative estimates of inpatient outcomes.

### Study population

We included adult patients (aged ≥18 years) hospitalized between 2016 and 2021 with a primary diagnosis of AIS identified using the International Classification of Diseases, Tenth Revision, Clinical Modification (ICD-10-CM) codes I63.x.x From this cohort, patients who had a concurrent diagnosis of STEMI during the same hospitalization were identified using the ICD−10-CM codes including I21.0, I21.1, I21.2, I21.3, I22.0, I22.1, I22.8 and I22.9. We excluded patients with non-ST elevation myocardial infarction (NSTEMI), intracranial hemorrhage, and those with missing data on key demographic and clinical variables.

### Outcomes

The primary outcome was in-hospital mortality. Secondary outcomes included the occurrence of acute kidney injury (AKI), cardiogenic shock, cardiac arrest, gastrointestinal (GI) bleeding, pericardial effusion, cardiac tamponade, cost of hospitalization and length of stay. In addition, we evaluated the trends of STEMI among AIS hospitalization as well as the trends of in-hospital mortality among AIS hospitalizations, stratified by presence of STEMI.

### Statistical analysis

All analyses accounted for the complex survey design using appropriate discharge-level weights to produce national estimates. Descriptive statistics were computed, with continuous variables presented as means (± standard deviations) and categorical variables as proportions. For comparisons between groups (AIS with and without STEMI), we used the chi-square test for categorical variables and the linear regression for continuous variables. Multivariable logistic regression models were implemented to assess the outcomes, adjusting for patient demographics, comorbidities, and hospital characteristics. Results were presented as adjusted odds ratios (ORs) with 95% confidence intervals (CIs). All statistical analyses were performed using Stata version 17.0 (StataCorp LLC, College Station, TX).

## Results

We evaluated 2,804,819 patients who presented to the hospital with Acute Ischemic Stroke. Of these patients who presented primarily for the AIS, 6,550 had a STEMI diagnosed during the hospitalization while 2,798,269 patients did not have a STEMI.

In the AIS population without concomitant STEMI, the average age was 70 years old and 49.78% of the cohort was female. In addition, the predominant race was white (68.14% of the population), followed by African American and Hispanic. In the AIS stroke that had concurrent STEMI, the average age was 70.48 years old, and 49.31% of the cohort was female. In addition, the predominant race was white (68.17% of the population) followed by African American and Hispanic.

Significant comorbidities that existed among both groups included heart failure, atrial fibrillation, valvular heart disease, hypertension, family history of ischemic heart disease, hyperlipidemia, hypothyroidism, liver disease, coagulopathy, obesity, fluid and electrolyte disorders, alcohol use, depression, prior percutaneous coronary intervention, prior myocardial infarction, prior stroke, cancer, obstructive sleep apnea (Table 1).

**Table 1:** Baseline characteristics of acute ischemic stroke patients with and without ST-elevation myocardial infarction (STEMI).

Variables	Without STEMI	With STEMI	*p*-value
Number of patients, *n*	2,798,269	6,550	-
Age	69.96 ± 13.88	70.48 ± 13.60	0.163
Female	1,392,964 (49.78)	3,230 (49.31)	0.7354
Race			0.9923
White	1,906,704 (68.14)	4,465 (68.17)	
Black	491,600 (17.57)	1,165 (17.79)	
Hispanic	230,800 (8.25)	510 (7.79)	
Asian or Pacific Islander	84,930 (3.04)	205 (3.13)	
Native American	12,835 (0.46)	30 (0.46)	
Other	71,400 (2.55)	175 (2.67)	
Hospital bed size			0.0031
Small	510,704 (18.25)	985 (15.04)	
Medium	817,969 (29.23)	1,850 (28.24)	
Large	1,469,596 (52.52)	3,715 (56.72)	
Hospital teaching status			<0.001
Rural	210,765 (7.53)	375 (5.73)	
Urban Non-teaching	561,795 (20.08)	985 (15.04)	
Urban Teaching	2,025,709 (72.39)	5,190 (79.24)	
Admission			0.1848
Elective	75,505 (2.70)	215 (3.28)	
Median household income, $			0.1251
1–28,999	868,155 (31.02)	1,910 (29.16)	
29,000–35,999	733,220 (26.20)	1,700 (25.95)	
36,000–46,999	661,625 (23.64)	1,525 (23.28)	
47,000+	535,270 (19.13)	1,415 (21.60)	
Hospital Region			0.0629
Northeast	487,180 (17.41)	1,240 (18.93)	
Midwest	594,535 (21.25)	1,435 (21.91)	
South	1,203,610 (43.01)	2,575 (39.31)	
West	512,944 (18.33)	1,300 (19.85)	
Primary payment			0.466
Medicare	1,807,464 (64.59)	4,225 (64.50)	
Medicaid	265,030 (9.47)	720 (10.99)	
Private insurance	535,700 (19.14)	1,185 (18.09)	
Self-pay	115,485 (4.13)	250 (3.82)	
No charge	8,500 (0.30)	25 (0.38)	
Other	66,090 (2.36)	145 (2.21)	
Comorbidities			
Congestive heart failure	498,785 (17.82)	3,080 (47.02)	<0.001
Atrial fibrillation	607,650 (21.72)	1,920 (29.31)	<0.001
Ventricular arrhythmias	43,685 (1.56)	655 (10.00)	<0.001
Valvular heart diseases	269,315 (9.62)	740 (11.30)	0.0447
Peripheral vascular disease	262,835 (9.39)	670 (10.23)	0.3019
Hypertension	2,383,669 (85.18)	5,270 (80.46)	<0.001
Diabetes mellitus	1,103,510 (39.44)	2,710 (41.37)	0.1462
Anemia	91,560 (3.27)	220 (3.36)	0.8597
Family history of IHD	157,415 (5.63)	280 (4.27)	0.0346
Hyperlipidemia	1,718,464 (61.41)	3,635 (55.50)	<0.001
Chronic lung disease	449,810 (16.07)	995 (15.19)	0.3839
Hypothyroidism	404,270 (14.45)	810 (12.37)	0.0341
CKD	520,710 (18.61)	1,335 (20.38)	0.0968
Liver disease	55,460 (1.98)	295 (4.50)	<0.001
Rheumatological disorders	79,575 (2.84)	130 (1.98)	0.0598
Coagulopathy	141,090 (5.04)	615 (9.39)	<0.001
Obesity	418,545 (14.96)	750 (11.45)	<0.001
Fluid and electrolyte disorders	652,305 (23.31)	2,445 (37.33)	<0.001
Alcohol abuse	128,490 (4.59)	195 (2.98)	0.0048
Depression	335,360 (11.98)	555 (8.47)	<0.001
Smoking	541,440 (19.35)	1,235 (18.85)	0.652
Prior MI	203,520 (7.27)	655 (10.00)	<0.001
Prior PCI	17,235 (0.62)	90 (1.37)	<0.001
Prior CABG	169,205 (6.05)	370 (5.65)	0.5427
Prior Stroke	439,220 (15.70)	705 (10.76)	<0.001
Cancer	115,535 (4.13)	545 (8.32)	<0.001
Obstructive Sleep Apnea	189,265 (6.76)	290 (4.43)	<0.001
Elixhauser comorbidities index	4.12 ± 2.00	5.02 ± 2.06	<0.001

**Figure 1:**
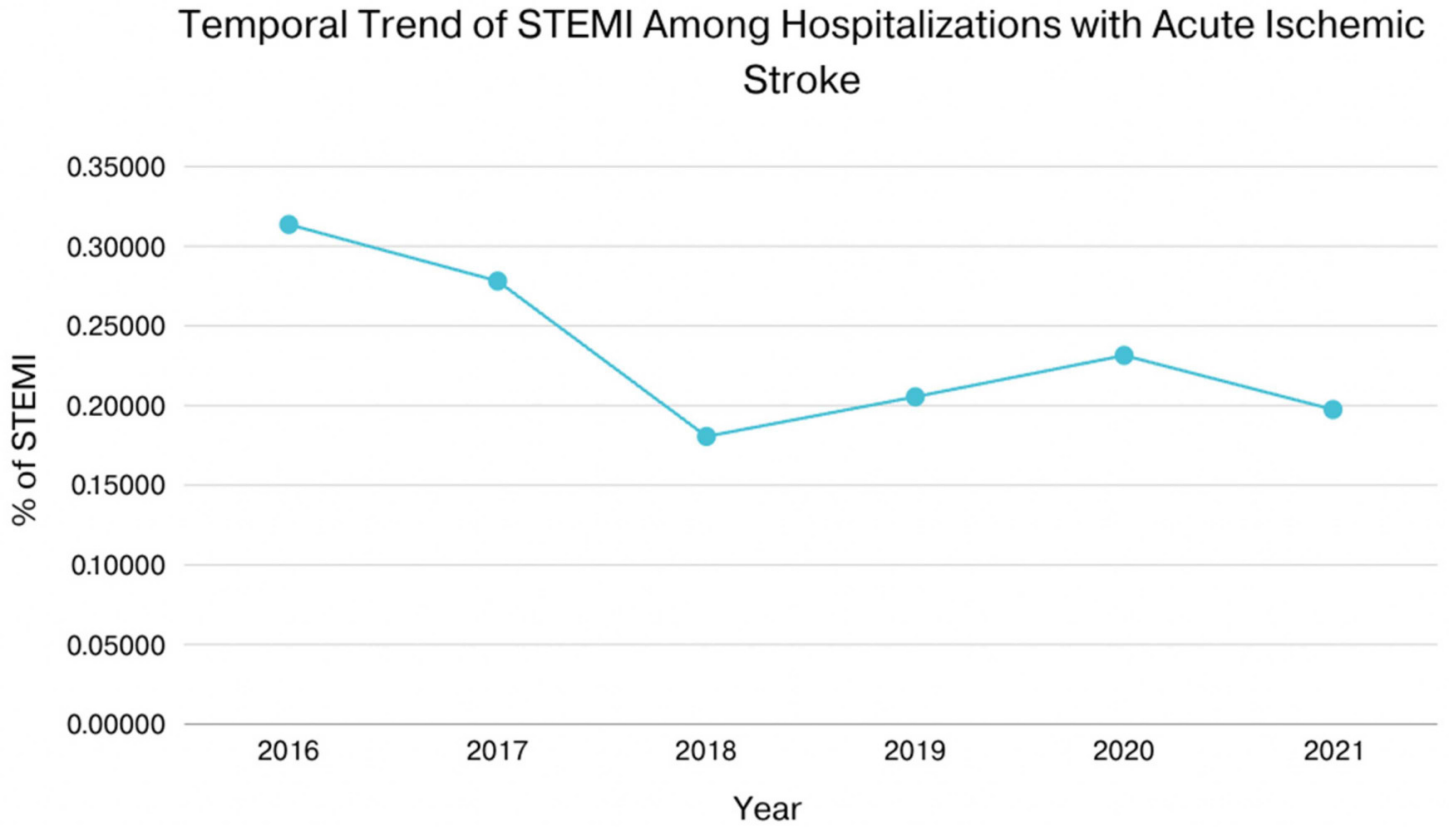
Shows that from 2016 to 2021, the yearly incidence of STEMI in this population has decreased from around 0.3% in 2016 to about 0.2% in 2021.

**Figure 2:**
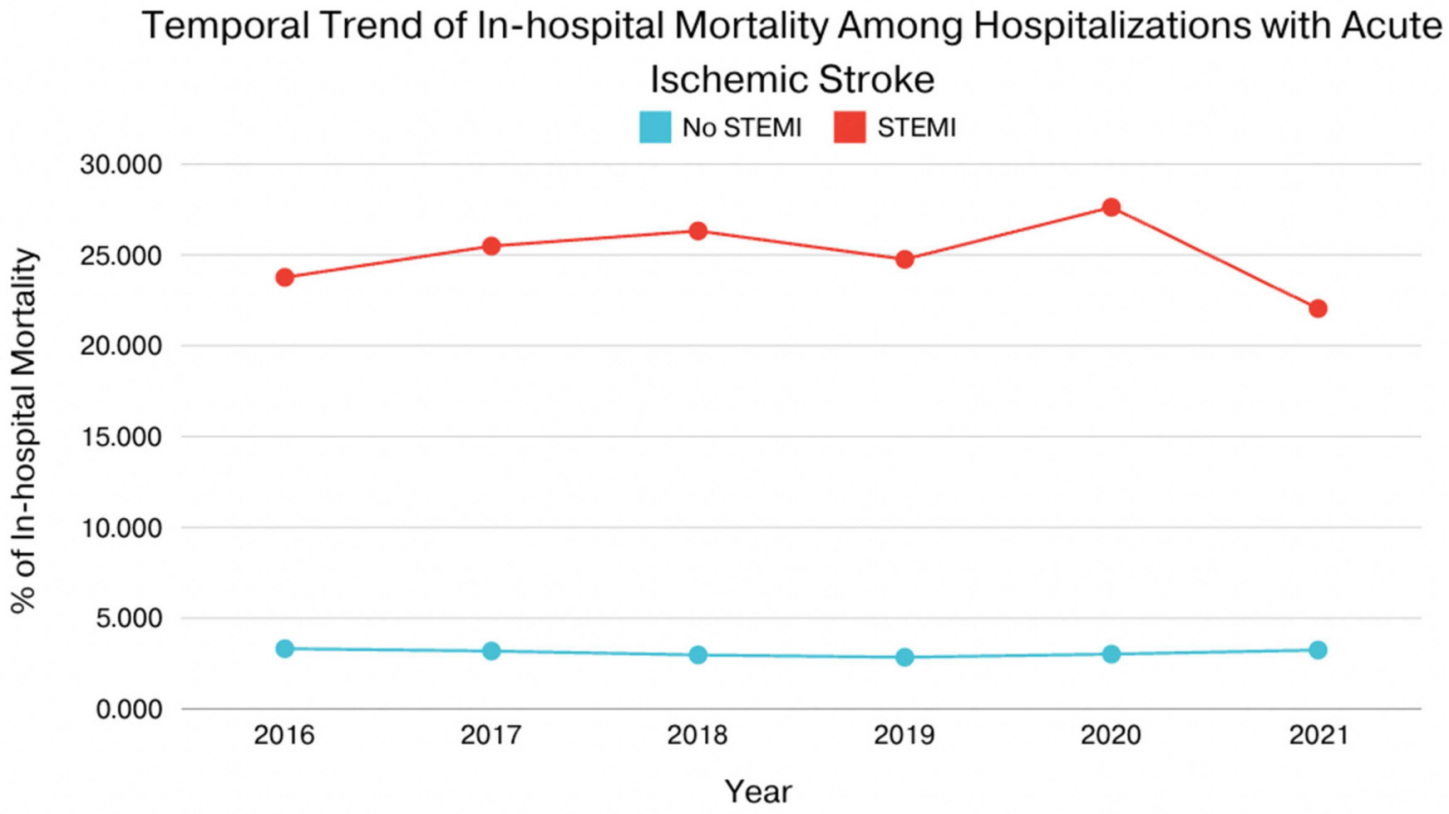
Compared mortality of hospitalizations with acute ischemic stroke stratified between those who had concomitant STEMI and those who did not.

In patients with AIS and concomitant STEMI, compared to those without STEMI, there were statistically significant differences in mortality, AKI, cardiac arrest, cardiogenic shock, GI bleeding, pericardial effusion, cardiac tamponade, septic shock, cost of hospitalization, and length of stay. The adjusted odds ratio for mortality was 7.43 (CI of 6.44–8.57 with *p*-value < .001), the adjusted odds ratio for AKI was 1.96 (CI of 1.72–2.23 with *p*-value < .001), the adjusted odds ratio for cardiac arrest was 7.76 (CI of 6.01–10.03 with *p*-value < .001), the adjusted odds ratio for cardiogenic shock was 29.64 (CI of 22.64–38.81 with *p*-value < .001). There was also a statistically significant difference in the need for procedures between the two groups in terms of requiring PCI, Fibrinolysis, mechanical intracranial thrombectomy, blood transfusion, use of mechanical circulatory support, ICD or pacemaker insertion, tracheostomy, and intubation (Tables 2, 3). We also sought to evaluate the yearly incidence of STEMI among those hospitalized with acute ischemic stroke between 2016 and 2021. Between 2016 and 2021, the incidence of STEMI among AIS admissions decreased from about 3 cases per 1,000 patients to about 2 cases per 1,000 patients and demonstrated a modest decline over the time. Figure 1 illustrates the annual incidence expressed as a percent of patients who had AIS with STEMI among all patients with AIS for each given year, and provides a more accurate depiction of trends in this population and shows that from 2016 to 2021, the frequency of STEMI in this population has decreased from around 0.30% in 2016 to about 0.20% in 2021. Furthermore, Figure 2 compared mortality of hospitalizations with acute ischemic stroke stratified between those who had concomitant STEMI and those who did not. It showed a significant difference with a higher mortality in the AIS with STEMI population compared to without STEMI.

**Table 2:** Procedures between acute ischemic stroke patients with and without ST-elevation myocardial infarction (STEMI).

Procedures	No STEMI	STEMI	Total	*p*-value
PCI including angiogram, angioplasty	13,925 (0.50)	1,025 (15.65)	14,950	<0.001
CABG	–	–	–	–
Fibrinolysis	267,925 (9.57)	770 (11.76)	268,695	0.0077
Mechanical intracranial thrombectomy	148,140 (5.29)	615 (9.39)	148,755	<0.001
Blood transfusion	34,615 (1.24)	260 (3.97)	34,875	<0.001
Use of mechanical circulatory support (LVAD, IABP, ECMO)	165 (0.01)	55 (0.84)	220	<0.001
ICD/pacemaker insertion	7,595 (0.27)	55 (0.84)	7,650	<0.001
Tracheostomy	12,765 (0.46)	130 (1.98)	12,895	<0.001
Intubation	80,615 (2.88)	1,230 (18.78)	81,845	<0.001

**Table 3:** Unadjusted and adjusted outcomes between acute ischemic stroke patients with and without ST-elevation myocardial infarction (STEMI).

In-hospital outcomes/complications	No STEMI	STEMI	Total	*p*-value	aOR^a^ (95% CI)	*p*-value
Mortality	86,610 (3.10)	1,635 (24.96)	88,245	<0.001	7.43 (6.44–8.57)	<0.001
Acute kidney injury	367,220 (13.12)	1,890 (28.85)	369,110	<0.001	1.96 (1.72–2.23)	<0.001
Cardiac arrest	12,135 (0.43)	370 (5.65)	12,505	<0.001	7.76 (6.01–10.03)	<0.001
Cardiogenic shock	3,390 (0.12)	545 (8.32)	3,935	<0.001	29.64 (22.64–38.81)	<0.001
Access site bleeding	3,375 (0.12)	20 (0.31)	3,395	0.0538	1.82 (0.68–4.88)	0.233
GI bleeding	23,215 (0.83)	170 (2.60)	23,385	<0.001	2.01 (1.42–2.84)	<0.001
Pericardial effusion	9,245 (0.33)	120 (1.83)	9,365	<0.001	3.46 (2.24–5.34)	<0.001
Cardiac tamponade	350 (0.01)	20 (0.31)	370	<0.001	11.27 (3.87–32.77)	<0.001
Septic shock	10,410 (0.37)	205 (3.13)	10,615	<0.001	3.38 (2.34–4.90)	<0.001
Cost of hospitalization	14,558.93 ± 15,743.11	24,932.65 ± 26,358.98	<0.001			
Length of stay	4.76 ± 6.18	7.30 ± 8.38	<0.001			

^a^ Adjusted for age, gender, race, hospital bed size, hospital teaching status, heart failure, atrial fibrillaiton, valvular heart disease, hypertension, family history of ischemic heart disease, hyperlipidemia, hypothyroidism, liver disease, coagulopathy, obesity, fluid and electrolyte disorders, alcohol use, depression, prior percutaneous coronary intervention, prior myocardial infarction, prior stroke, cancer, obstructive sleep apnea.

## Discussion

The pathogenesis of acute ischemic stroke (AIS) and ST segment elevation myocardial infarction (STEMI) share several similarities, primarily characterized by ischemic etiology and inflammatory processes. Both conditions result from a significant reduction in blood flow due to the obstruction of blood vessels; STEMI typically occurs due to rupture of an atherosclerotic plaque and subsequent thrombus formation in coronary arteries, while AIS may arise from thromboembolic occlusion of cerebral arteries or large vessel disease. In both scenarios, inflammatory processes, platelet activation, and coagulation play critical roles in thrombus formation, contributing to tissue damage. However, key differences exist between the two conditions. Notably, the affected anatomical regions differ, with STEMI impacting the heart and AIS affecting the brain, which has profound implications for tissue response and recovery mechanisms. Additionally, the underlying causes of each condition can vary. While both conditions can arise from atherosclerosis, STEMI is more frequently associated with acute plaque rupture and thrombosis, whereas AIS can also occur due to systemic factors like atrial fibrillation or carotid artery disease, leading to embolic strokes (1) Treatment strategies also differ significantly; STEMI is primarily managed through percutaneous coronary intervention or thrombolytics, AIS often requires thrombolysis or mechanical thrombectomy. Recent multicenter studies have further refined predictors of poor outcomes and hemorrhagic transformation following alteplase in AIS, underscoring the importance of individualized risk assessment in this setting (2, 3). These findings reinforce that while thrombolysis remains a cornerstone of AIS management, its risks, particularly in patients with atrial fibrillation or concomitant cardiac disease, necessitate careful patient selection and multidisciplinary decision-making. The time sensitivity for intervention varies. While both conditions are considered acute emergencies, the brain is less tolerant of ischemia compared to the heart, necessitating faster intervention for AIS to minimize irreversible damage. Although the pathogenesis of both acute ischemic stroke and STEMI are often similar and linked to parallel factors, there is not significant data studying and managing patients admitted for stroke who then experience a STEMI.

There is also a mechanistic link between AIS and STEMI. The occurrence of STEMI in patients hospitalized with AIS may be explained through several interconnected mechanisms collectively described as the *stroke-heart syndrome* (4) acute brain injury can trigger autonomic dysregulation with excessive sympathetic activation and parasympathetic withdrawal, resulting in a catecholamine surge that predisposed to myocardial ischemia, arrhythmia, and contractile dysfunction. This neurocardiogenic injury is further compounded by systemic inflammatory responses and endothelial dysfunction which amplify pro-thrombotic pathways and microvascular damage (5). In addition, stroke itself promotes a transient hypercoagulable state through platelet activation, increasing circulating clotting factors, and impaired fibrinolysis, therapy increasing the risk of coronary artery thrombosis (6). Finally, AIS patients frequently have concomitant atherosclerotic disease, which, in the setting of heightened hemodynamic stress and metabolic demand, may destabilize vulnerable coronary plaques and precipitate acute coronary occlusion (7–9). Together, these mechanisms provide a pathophysiological framework that explains the markedly higher rates of myocardial infarction, cardiogenic shock, and mortality observed in our cohort of AIS patients with STEMI (10, 11).

In 2003, Adam et al. (12) described coronary risk evaluation in patients with TIA and ischemic stroke. Given that stroke and myocardial infarction share common risk factors and pathological mechanisms, coronary artery disease is an important cause of death in patients with cerebrovascular disease. This Scientific statement was made to highlight issues in management of CAD patients with brain ischemia who do not have previously identified CAD, and provide recommendations for further research to determine the optimal approach to recognize and treat asymptomatic CAD in patients with cerebral ischemia. This was a stepping stone in increasing research in not just CAD but also incidence and treatment of STEMI in cerebrovascular patients.

Amarenco et al. (7) describes coronary artery disease and risk of major vascular events after cerebral infarction. This was the first study to correlate angiographic findings with subsequent cardiovascular events in patients with cerebral infarction. In patients with cerebral infarction, asymptomatic CAD is highly predictive of future major cardiovascular events.

These studies were also frameworks for further investigation that focused not only on CAD, but also on myocardial infarctions. Toueze and colleagues (13) performed a meta-analysis and systematic review to determine the risk of MI and nonstroke vascular death after TIA, utilizing 39 cohort studies with a total of 65,996 patients. Through previous analyses, it was found that compared with the general population, stroke patients have an increased risk of death that notably results from myocardial infarction. But there was no reliable estimation of the absolute risk of MI and vascular death after stroke and high risk populations. This systemic review and meta-analysis aimed to study the absolute risk of MI and vascular death after stroke or TIA. The main result of this meta-analysis is that after a stroke or a TIA, the risks of MI and nonstroke vascular death are each about 2% per year, which is usually considered high absolute risks in different guidelines for assessment of cardiovascular risk. Given that patients with TIA or stroke have a relatively high risk of MI and nonstroke vascular death, it leads us to where further research may be needed such as the need to identify the determinants of coronary artery disease in stroke patients. It also urges us to improve secondary prevention. These can be in the form of preventive measures without CAD screening, systematic screening of asymptomatic patients, selective screening based on risk stratification, while also being conscious of the downfalls of over-screening. Further research to identify and address factors that lead to risk of CAD and address the best possible interventions to decrease both cardiac morbidity and mortality in stroke patients.

In 2017, in Alqahtani et al. (14) studied the incidence and outcomes of myocardial infarction in patients admitted with ischemic stroke. They utilized the National Inpatient Sample to identify patients with AIS between 2003 and 2014 who were then analyzed for incidence of MI and in hospital mortality. They found that AMI after a stroke carried a substantial in-hospital mortality and cost of care and that although coronary angiography was utilized in a minority of these patients, there may be improved survival in those who underwent coronary angiography with or without intervention. They suggested further studies were needed to discern the ideal approach to addressing STEMI in AIS patients.

Our data seems to be congruent with the results of Alqahtani et al. and Amerenco et al. in which we found a significant mortality increase in those admitted with AIS who experienced STEMI in comparison to those who did not have a STEMI. There also was a significant improvement in hospital mortality with those who ended up undergoing cardiac catheterization. The studies differed in that Al Qahtani et al. reported an increase in myocardial infarction over their studied time interval, however, this was mainly driven by an increase in diagnosis of NSTEMI's whereas our study showed a decrease in myocardial infarction, and focused particularly on STEMI's.

However, there were also several limitations of the study. First, the National Inpatient Sample (NIS) is a discharge-level administrative database without patient identifiers; therefore, repeat hospitalizations for the same individual cannot be distinguished. Second, important clinical details that influence outcomes in acute ischemic stroke (AIS) are not available, including baseline modified Rankin Scale (mRS), National Institutes of Health Stroke Scale (NIHSS) scores when not coded, TOAST classification, vascular territory, blood pressure trajectories, temperature at presentation, and door-to-needle times. Third, while we report rates of fibrinolysis and mechanical thrombectomy, the NIS does not capture the specific thrombolytic agent (e.g., alteplase vs. tenecteplase), the timing of administration, or subsequent hemorrhagic complications. Thus, not separating acute ischemic stroke by severity which is a key confounder. Fourth, as with all administrative datasets, diagnoses and procedures are subject to potential coding misclassification. However, prior validation studies have demonstrated high accuracy for both ischemic stroke and STEMI codes in ICD-10. Despite these limitations, the large, nationally representative sample and robust associations observed strengthen the validity and clinical relevance of our findings.

In conclusion, our study highlights the incidence of STEMI in patients already hospitalized with AIS, risk factors that increase the risk, and the outcomes and complications that tend to arise after experiencing a STEMI after a stroke. This dual pathology requires an integrated approach to management that addresses the complexities of treating concurrent ischemic events.

The question of whether cardiac screening should be intensified in AIS has been increasingly recognized within the broader concept of stroke-heart interactions. Current AHA/ASA stroke guidance recommends obtaining a 12-lead ECG and baseline cardiac troponin at admission, along with continuous in-hospital rhythm monitoring to detect atrial fibrillation and malignant arrhythmias. In patients with symptoms, ischemic-type ECG changes, or dynamic troponin elevation, serial ECG's and repeat troponin testing over the first 24–48 h are advised, ideally with early cardiology involvement. For secondary prevention, particularly in cryptogenic stroke, extended outpatient rhythm monitoring, including long-term patch monitoring or implantable loop recorders, is supported because it significantly improves atrial fibrillation detection and alters management. While stroke-specific ACS algorithms are lacking, contemporary cardiology guidelines provide a framework for management when STEMI is suspected during AIS hospitalization. This includes rapid ECG acquisition, serial high-sensitivity troponin testing, and multidisciplinary decision-making to balance urgent revascularization with the elevated risk of intracranial bleeding after thrombolysis or thrombectomy. Recent guidance now further emphasizes risk-adapted strategies: the 2024 ACC Expert Consensus Pathway recommends tailoring arrhythmia monitoring such as short-term event monitor, patch, or implantable devices according to stroke subtype and embolic risk, while the 2025 ACC/AHA ACS guideline highlights the importance of serial high-sensitivity troponin testing and structured multidisciplinary care pathways when ACS is suspected during AIS hospitalization (15, 16). Collectively, these recommendations support a selective, risk-based intensification of cardiac monitoring rather than a universal escalation for all AIS patients.

Future research should focus on elucidating the underlying mechanisms that predispose stroke patients to STEMI, as understanding these pathways may inform targeted therapies and preventive strategies. Furthermore, establishing clear protocols for the early identification and management of STEMI in hospitalized AIS patients is essential. Medicinal therapeutic approaches, such as the dual antiplatelet therapy or novel anticoagulants warrant exploration in this unique patient population to reduce the risk of recurrent events. Additionally, the development of interdisciplinary care teams that include neurologists, cardiologists, and rehabilitation specialists could facilitate comprehensive treatment plans and improve overall outcomes. As the field of cardiovascular and cerebrovascular medicine continues to evolve, ongoing clinical trials are necessary to evaluate the efficacy and safety of various interventions either medical or surgical, ultimately aiming to enhance patient care and reduce mortality and morbidity in this vulnerable group. Addressing the intersection of AIS and STEMI will be crucial for optimizing the management of these patients and improving long term outcomes in those who unique challenges faced by individuals experiencing concurrent ischemic events.

## Data Availability

The original contributions presented in the study are included in the article/Supplementary Material, further inquiries can be directed to the corresponding author.
